# Artificial intelligence-enhanced care pathway planning and scheduling system: content validity assessment of required functionalities

**DOI:** 10.1186/s12913-022-08780-y

**Published:** 2022-12-12

**Authors:** Miia Jansson, Pasi Ohtonen, Timo Alalääkkölä, Juuso Heikkinen, Minna Mäkiniemi, Sanna Lahtinen, Riikka Lahtela, Merja Ahonen, Sirpa Jämsä, Janne Liisantti

**Affiliations:** 1 Research Unit of Medical Imaging, Physics and Technology, University of Oulu, Oulu, Finland; 2 Research Unit of Surgery, Anesthesia and Intensive Care, Oulu University Hospital, University of Oulu, Oulu, Finland; 3 Testing and Innovations, Oulu University Hospital, Oulu, Finland; 4 Division of Orthopedic and Trauma Surgery, Department of Surgery, Medical Research Center, Oulu University Hospital, Oulu, Finland; 5 Oulu University Hospital, Oulu, Finland; 6 Department of Anesthesiology, Oulu University Hospital, Oulu, Finland; 7 MRC Oulu, Research Group of Anesthesiology, Oulu, Finland; 8 Sense Organ Diseases Centre, Oulu University Hospital, Oulu, Finland

**Keywords:** AI, Human-in-loop, human-AI interaction, Machine learning, Care pathway

## Abstract

**Background:**

Artificial intelligence (AI) and machine learning are transforming the optimization of clinical and patient workflows in healthcare. There is a need for research to specify clinical requirements for AI-enhanced care pathway planning and scheduling systems to improve human–AI interaction in machine learning applications. The aim of this study was to assess content validity and prioritize the most relevant functionalities of an AI-enhanced care pathway planning and scheduling system.

**Methods:**

A prospective content validity assessment was conducted in five university hospitals in three different countries using an electronic survey. The content of the survey was formed from clinical requirements, which were formulated into generic statements of required AI functionalities. The relevancy of each statement was evaluated using a content validity index. In addition, weighted ranking points were calculated to prioritize the most relevant functionalities of an AI-enhanced care pathway planning and scheduling system.

**Results:**

A total of 50 responses were received from clinical professionals from three European countries. An item-level content validity index ranged from 0.42 to 0.96. 45% of the generic statements were considered good. The highest ranked functionalities for an AI-enhanced care pathway planning and scheduling system were related to risk assessment, patient profiling, and resources. The highest ranked functionalities for the user interface were related to the explainability of machine learning models.

**Conclusion:**

This study provided a comprehensive list of functionalities that can be used to design future AI-enhanced solutions and evaluate the designed solutions against requirements. The relevance of statements concerning the AI functionalities were considered somewhat relevant, which might be due to the low level or organizational readiness for AI in healthcare.

## Background

Artificial intelligence (AI) and machine learning (ML) are transforming the optimization of clinical and patient workflows in healthcare. The adoption of AI and ML technologies in care pathway planning and scheduling systems can enable early risk assessment [1], provide more accurate schedules [2–7], reduce blocking [8], and thus, maximize efficiency [9], minimize unnecessary costs [10], and tackle excessive waiting times [11] throughout the care pathway. However, the current care pathway planning and scheduling systems are mostly manual, time-consuming, and resource intensive [8]. In addition, resource allocation in healthcare seems to be backwards looking and based on prior caseloads.

Due to growing demand for healthcare services [12], there is a great need for advanced care planning. Intelligent digital services are usually approached using mathematical modeling and made available to users through dedicated software. Yet, despite its clinical potential, AI is not a universal solution. Uncertainty, organizational readiness, and workflow integration have been the major barriers toward the widespread adoption of medical AI [13, 14]. There is a need for research to specify clinical requirements for an AI-enhanced care pathway planning and scheduling system to improve human–AI interaction in ML applications [15].

Human-centered methods can be used to identify end-users’ needs for AI-based clinical decision support systems. According to the ISO 9241 − 210 [16], “*Human-centered design is an approach to interactive systems development that aims to make systems usable and useful by focusing on the users, their needs and requirements, and by applying human factors/ergonomics, and usability knowledge and techniques*”. The ISO framework of human-centered design includes interactive and iterative phases to understand and specify the context of use, specify user requirements, design a solution, and evaluate the design against requirements.

This study is a part of a larger research and development project that develops existing digital solutions further together with hospitals, technology providers, and researchers (https://aiccelerate.eu/). This article, however, focuses solely on specified user requirements to assess content validity and prioritize the most relevant functionalities of an AI-enhanced care pathway planning and scheduling system at the patient, unit, and resource levels.

## Methods

### Study design

A cross-sectional survey was carried out to assess content validity and prioritize the most relevant functionalities of the AI-enhanced care pathway planning and scheduling system. All methods were carried out in accordance with relevant guidelines and regulations (Declaration of Helsinki 2013).

### Sample

Prospective content validity assessment was conducted in five university hospitals (Sant Joan de Déu Barcelona Children’s Hospital, Bambino Gesù Children’s Hospital, Department of Neuroscience of the University of Padua, and Helsinki University Hospital) in three different countries (incl. Finland, Italy, and Spain) included in the AICCELERATE consortium between October 11th 2021 February and November 30th April 2021. The study protocol was approved by the clinical partners of the AICCELERATE Project Consortium and by the local Institutional Review Board of Oulu University Hospital (Ref no. 98/2022).

### Study procedures

This study was conducted in two phases: (1) domain identification, item generation, and survey formation and (2) content validation and content prioritization.

#### Phase 1: Domain identification, item generation, and survey formation

The content of the survey was formed from clinical requirements, which were collected from three AICCELERATE Smart Hospital Care Pathway Engine pilots from beforementioned countries (https://aiccelerate.eu/) by using human-centered methods such as solution charts, user personas, blueprints, and UI-sketches of the solution. These pilots focused on (1) patient flow management for surgical units (Pilot 1); (2) digital care pathway for Parkinson’s disease (Pilot 2); and (3) pediatric service delivery (Pilot 3). Clinical requirements were then formulated into generic statements of functionalities and grouped into seven categories:

1. The first category covered factual data on demographics (e.g., country, age, gender, work tenure, profession). In addition, one question related to information and decision-making (“*Which of the following best describes how to use the information and knowledge to support your own work*”) with 6 response alternatives was included in baseline characteristics.
2. The second category covered self-report statements regarding the relevancy of unit-level recommendations for operation on a 4-point Likert scale (1, not relevant; 2, somewhat relevant; 3, quite relevant; 4, highly relevant).
3. The third category covered self-report statements regarding the relevancy of unit-level recommendations for patient predicted perioperative processes/patient flows on a 4-point Likert scale (1, not relevant; 2, somewhat relevant; 3, quite relevant; 4, highly relevant).
4. The fourth category covered self-report statements regarding the importance of patient-level functionalities in the user interface (UI) on a 4-point Likert scale (1, not important; 2, somewhat important; 3, quite important; 4, highly important).
5. The fifth category covered self-report statements regarding the importance of unit-level functionalities in the UI on a 4-point Likert scale (1, not important; 2, somewhat important; 3, quite important; 4, highly important).
6. The sixth category covered self-report statements regarding the importance of functionalities in the UI (at the unit resource level) on a 4-point Likert scale (1, not important; 2, somewhat important; 3, quite important; 4, highly important).
7. The last category contained an open text field to invite the respondent to leave open-ended comments and suggest additional features that were missing from the statements (“*Can you think of any other functions that would be necessary for the upcoming system? If so, please specify*”).

After domain identification, item generation, and instrument formation (highly structured, self-administered, multiple-choice questionnaire), six experts (2 anesthesiologists, 2 registered nurses, 1 ICT support specialist, 1 biostatistician) evaluated the relevance, accuracy, clarity, and readability of each statement and identified whether any important issues were lacking. Based on the experts’ suggestions, minor revisions were made to the instructions, wording, and content of the survey.

A link to the questionnaire was sent to a contact person in each of five participating university hospitals via email. When the link to the questionnaire was sent to the local contact persons, they were instructed to share the link to suitable experts working at their hospitals (purposive sampling). The email included a brief introductory letter about the current status of healthcare systems and the utilization of AI-enhanced solutions, goals of the AICCELERATE project, progress of the project thus far, and the importance of participation in the survey. The response time was initially one week. Due to the low number of responses, the response time was eventually extended to seven weeks, and the respondents were reminded three times. The completion of the survey took approximately 10‒50 min.

#### Phase 2: Content validation and prioritization

The content validity was assessed following a structured procedure by an expert panel comprising clinical professionals, who were selected for their methodological and/or clinical expertise. Following Lynn [17], the respondents were asked to estimate the relevance of each generic statement independently on a 4-point Likert scale. As an additional indicator of relevancy, the respondents were asked to prioritize the importance of each generic statement independently on a 5-point scale (from the 1st to 5th most important). The respondents were also encouraged to give open comments and explain additional clinical requirements at the end of the survey.

### Data analysis

A content validity assessment was applied to evaluate the relevance of the content. An item-level content validity index (I-CVI) was calculated by dividing the number of responders rating the item as quite or highly relevant by the total number of respondents that gave an acceptable rating. An I-CVI of > 0.83 was considered good [17]. Additionally weighted ranking points (WRP) were calculated: the respondents were asked to rate five (four in categories 4, 5 and 6) most important statements. We recoded the first, second, and third ranked statements by 60, 30, and 10, respectively, to emphasize the differences in importance between the first, second, and third-ranked statements. Finally, the WRP was calculated from the sum of the recoded values [18]. Due to low number of open-ended comments these were not analyzed.

## Results

The final survey contained 6 items and 33 statements divided into seven main categories: demographics (6 items), relevancy of unit-level recommendations for operation (13 statements), relevancy of unit level recommendations for patients (6 statements), the importance of patient-level functionalities in the UI (5 statements), the importance of unit level functionalities in the UI (5 statements), and the importance of functionalities in the UI (4 statements).

### Category 1: demographics

The majority of the respondents (*n* = 50) were Finnish (30, 60.0%) and female (37, 75.5%) (Table 1). Most of respondents (31, 62.0%) used multiple information systems for retrospective analytics (Table 2). However, a minority (2, 4.0%) had systems for predictive analytics.

**Table 1:** Baseline characteristics of respondents

Characteristics (*n* = 50)	n (%)
Country	
Finland	30 (60.0)
Italy	9 (18.0)
Spain	11 (22.0)
Age	
< 29 years	2 (4.0)
30–39 years	17 (34.0)
40–49 years	18 (36.0)
50–59 years	9 (18.0)
> 60 years	4 (8.0)
Gender	
Female	37 (75.5)
Male	12 (24.5)
Work tenure	
< 1 year	0 (0.0)
1–5 years	3 (6.0)
6–10 years	12 (24.0)
11–20 years	17 (34.0)
21–30 years	12 (24.0)
> 30 years	6 (12.0)
Profession	
Chief physician	9 (18.0)
Chief nurse	4 (8.0)
Physician	16 (32.0)
Nurse	13 (26.0)
Other	8 (16.0)

**Table 2:** The use the information technology to support decision-making in a daily practice

Response alternatives		n (%)
**Reporting**:	I have manual reports available to see what the case/situation is (what happened)	12 (24.0)
**Dashboards**:	I have multiple information systems and a lot of data that I can individually use to analyze and measure the case/situation (why did it happen)	31 (62.0)
**Predictions**:	My work and decision-making are supported by smart hospital systems that merge data from multiple data sources and give predictions, recommendations and/or measurable results to anticipate the future (what will happen)	2 (4.0)
**Prescriptive**:	I have utilized smart hospital systems to augment and complement my human work and decision-making process with consistent and measurable results (how can we make it happen)	1 (2.0)
**Innovations**:	I am creatively utilizing, innovating and developing use of smart hospital systems and finding new ways to stay a step ahead in patient-centric care	3 (6.0)
**None of the above**		1 (2.0)

### Category 2: Relevancy of unit-level recommendations for operation

The top three ranked statements were: (1) It is important that AI recognizes if the patients are in risk for adverse events during the care; (2) It is important that AI is able to make individual patient profiles based on previous data; and (3) It is important that AI can suggest the best possible timing for a treatment or visit based on patient risks and the predicted patient flow. The ranking within the category as well as overall ranking can be seen in Table 3.

### Category 3: Relevancy of unit-level recommendations for patient-predicted perioperative processes/patient flows

The top three ranked statements were: (1) It is important that AI is able to recognize the possible factors and patterns causing adverse events after care or prolonged need for care; (2) It is important that AI is able to predict available resources for certain time points based on data of internal and external factors; and (3) It is important that AI is able to recognize the days of increased need for care and increased need for resources and the factors causing them. The ranking within the category as well as overall ranking can be seen in Table 3.

### Category 4: Importance of patient-level functionalities in the UI

The top three ranked statements were: (1) The user interface updates the visualization of the predicted evolution of the patient’s condition based on historical and live patient data; (2) The user interface has a visualization for the predicted patient flow and the reasoning behind it for a particular patient; and (3) The user interface has functionalities for finding the appropriate and right timing for a particular patient’s treatment. The ranking within the category as well as overall ranking can be seen in Table 3.

**Table 3:** Relevancy of an AI-enhanced care pathway planning and scheduling system functionalities

Statements		I – CVI	WRP (ranking withing the category)
Relevancy of unit-level recommendations for operation.	1. It is important that AI is able to make individual patient profiles based on previous data	0.82	2
	2. It is important that AI can suggest the best possible timing for a treatment or visit based on patient risks and predicted patient flow	0.9	3
	3. It is important that AI recognizes if the patients are in risk for adverse events during the care	0.96	1
	4. It is important that AI takes into account uncontrollable/external factors (weather, major events, holidays) while scheduling for the care	0.42	9
	5. It is essential that AI identifies the most relevant examinations and tests when planning a treatment event	0.86	5
	6. It is important that AI is able to recognize unnecessary laboratory tests for individual patients for the treatment/care	0.66	13
	7. It is important for AI to identify patient level patterns that cause last minute cancellation of treatment/care	0.72	11
	8. AI must estimate the duration of all phases of treatment events	0.54	10
	9. AI idenfies and takes into account adverse or atypical events when predicting the care pathway and duration of its phases.	0.71	12
	10. It is important that AI recommends the best time for the planned treatment for the patient, taking into account other scheduled patients, patient flow and available resources.	0.84	7
	11. It is important for AI to identify patterns from the live data that predicts deterioration in the patient`s chronic diseases.	0.82	6
	12. It is important that AI is able to use data from home sensors, wearables and robots when predicting individual patient’s symptom progression	0.76	8
	13. It is important that AI recognizes patterns from patient data that are associated with deterioration during the following 24 h	0.88	4
Relevancy of unit-level recommendations for patients predicted perioperative process/patient flows	1. It is important that AI is able to predict available resources for certain time points based on data of internal and external factors	0.86	2
	2. It is important that AI is able to estimate the needed time resources for the scheduled day.	0.84	4
	3. It is important that AI is able to recognize the possible factors and patterns causing adverse events after care or prolonged need for care	0.82	1
	4. It is important that AI is able to recognize the days of increased need for care and increased need for resources (“high flow days”) and the factors causing those	0.88	3
	5. It is important that AI is able to predict the days of decreased personnel resources in care	0.76	5
	6. It is important that AI is able to predict the average duration of provided care	0.76	6
Importance of patient-level functionalities in the UI	1. The user interface has a visualization of predicted patient flow and a reasoning behind it for a particular patient (predicted duration of each phase of his/her care path)	0.78	2
	2. The user interface has functionalities for finding an appropriate and right timing for a particular patient’s treatment	0.88	3
	3. The user interface updates the visualization of the patient flow for a particular patient during care based on existing data enriched with live data	0.73	4
	4. The user interface has a report/list of the patients whose treatment is anticipated to be at risk for cancellation	0.66	5
	5. The user interface updates the visualization of predicted evolution of patient’s condition based on the historical and live patient data	0.84	1
Importance of unit-level functionalities in the UI	1. The user interface has a visualization of units/hospitals general patient flow (all predicted patients flows)	0.72	3
	2. The user interface updates the visualization of the patient flow for a particular patient during care based	0.76	2
	3. The user interface has a view of the recommended order of patients’ treatment	0.82	1
	4. The user interface has a report/list of similar patients to replace cancelled ones	0.70	4
	5. The user interface has a view of predicted changes in the patient flow based on uncontrollable factors like weather, major events and holiday seasons	0.54	5
Importance of functionalities in the UI (unit resources)	1. The user interface has a functionality to check if staff availability is anticipated to be limited during planned treatment time	0.78	1
	2. The user interface has a functionality to check if hospital capacity is anticipated to be limited during plan planned treatment time	0.86	2
	3. The user interface has a visualization of predicted hospital capacity as a replicate of hospital environment (i.e. digital twin)	0.56	3
	4. The user interface has a report/list of predicted financial impacts of the clinical care process	0.44	4

### Category 5: Importance of unit-level functionalities in the UI

The top three ranked statements were: (1) The user interface has a view of the recommended order of patient treatment; (2) The user interface updates the visualization of the patient flow for a particular patient during care; and (3) The user interface has a visualization of the general unit/hospital patient flow. The ranking within the category as well as overall ranking can be seen in Table 3.

### Category 6: Importance of functionalities in the UI

The top three ranked statements were: (1) The user interface has a functionality to check if limited staff availability is anticipated during the planned treatment time; (2) The user interface has a functionality to check if the hospital capacity is anticipated to be limited during the planned treatment time; and (3) The user interface has a visualization of the predicted hospital capacity as a replicate of hospital environment. The ranking within the category as well as overall ranking can be seen in Table 3.

### Data summary

In general, the I–CVIs ranged from 0.42 to 0.96, and the average CVI was 0.754. 45% of the generic statements were considered good. According to the WRPs, the highest ranked functionalities for the AI-enhanced care pathway planning and scheduling system were related to risk assessment, patient profiling, and resources (Table 4). Correspondingly, the highest ranked functionalities for UI were related to the explainability of ML models.

**Table 4:** Summary of the results

The highest ranked statements	I – CVI
It is important that AI recognizes if the patients are in risk for adverse events during the care	0.96
It is important that AI is able to predict available resources for certain time points based on data of internal and external factors	0.86
The user interface updates the visualization of predicted evolution of patient’s condition based on the historical and live patient data	0.84
The user interface has a view of the recommended order of patients’ treatment	0.82
The user interface has a functionality to check if hospital capacity is anticipated to be limited during plan planned treatment time	0.86

## Discussion

According to our findings, the highest ranked functionalities for AI-enhanced care pathway planning and scheduling systems were related to risk assessment, patient profiling, and the use of shared resources (e.g., personnel, time) at the patient and unit levels. In the literature, AI-enhanced scheduling systems have been used to identify modifiable risk factors and to stratify patients into high- and low-risk groups to optimize preventive measures in advance [1, 19, 20]. In addition, intelligent digital services have been used to predict the duration of surgery (DOS) [2–7] and the postoperative length of stay [2] to optimize resource management with a high degree of accuracy.

The highest ranked functionalities for the UI were related to the explainability of ML models (e.g., predictors, visualization) which is line with newly adopted European Medical Device Regulation (EU 2017/745) [21], the upcoming EU AI Act (2021/0106/COD) [22], and the initiative Digital Health Software Pre-certification (Pre-Cert) Program [23]. In general, uncertainty and distrust of AI predictions have been the major barriers toward the widespread adoption of medical AI [13]. This mistrust is often due to the shortage of model explainability, where the relationship between the input and output of the underlying algorithms is unclear [24].

In addition, many organizations are still unfamiliar with digital transformation due to organizational (e.g., motivational readiness, institutional resources, staff attributes, and organizational climate) [14], technical (e.g., limited technology capabilities), and non-technical (e.g., lack of management support) challenges [25]. In this regard, the organization’s readiness for the adoption of AI is critical to the success of technological change. According to Jöhnk et al. [25], possible application scenarios of AI are not always directly obvious, and organizations must understand the technology to decide on the intended adoption purpose. For that reason, organizations must continuously assess and develop their AI readiness including strategic alignment (AI-business potentials, customer AI readiness), resources (e.g., financial budget, IT infrastructure), knowledge (e.g., AI awareness, upskilling, AI ethics), culture (e.g., change management, innovativeness), and data (e.g., availability, quality) in the AI adoption process to ensure its successful integration and avoid unnecessary investments and costs [14, 25].

In this study, the most relevant functions were related to situational awareness (e.g., the risk of adverse effects, clinical deterioration, or triage) instead of optimal resource usage (e.g., cancellations, overstays, unnecessary laboratory tests etc.) or organizational necessity highlighting both context- and purpose-specific perspectives on AI readiness. In the previous literature, user perceptions toward digital transformation have varied between professional groups, demonstrating the different needs and expectations associated with specific roles and responsibilities [26].

The obtained results of this study highlight the preoperative phase of the surgical path (e.g., personalized risk assessment and optimization). It must be noted, however, that intra- (e.g., the actual DOS) and postoperative phases (e.g., early detection of adverse effects/events) are equally important for the continuum and coordination of care to improve the workflow and reduce blocking, for instance. In addition, explainable AI could also be used to facilitate shared decision-making by helping patients to understand their individual risks and outcomes to select the available treatment options according to individual needs and goals [25]. However, the current use of information systems seems to be backwards looking.

Improving trust requires the development of more transparent ML methods in the near future. In fact, human-AI interaction is warranted to improve transparency in medical AI and thus, support accurate and trustable decision-making [15]. In addition, the expertise of respondents as well as novel research methods should be taken into account. Despite its widespread lack of familiarity, the future of AI is promising. Novel methods are needed to identify “unknown unknowns” in innovative projects.

### Limitations

Our study had several limitations related to sampling, participation, and response bias. First, the sample size was limited, but still covered five university hospitals in three different countries. In addition, the response rate of the selected experts was not calculated. Second, the majority of the respondents were physicians. In addition, most of respondents were from Finland, which may have affected the perceived relevance. The survey was however sent to all suitable experts, including all professions. In addition, repeated reminders of the survey were sent by the local contact persons. We were, however, unable to control multiple submissions (if any) and unintended respondents. Third, response bias may also have had an impact on the validity of survey. This kind of research bias was minimized by conducting the survey anonymously. Fourth, the relevance of statements concerning AI functionalities was considered somewhat relevant. This might be due to the low level of organizational readiness for AI in healthcare.

## Conclusion

This study provided a comprehensive list of functionalities that can be used to design future AI-enhanced solutions and evaluate the designed solutions against requirements. The relevance of statements was considered somewhat relevant, which might be due to the low level of organizational readiness for AI in healthcare.

## Data Availability

The datasets generated and analyzed are not publicly available. Datasets are available from the corresponding author on reasonable request and with permission from the relevant academic center.

## Abbreviations

AI: Artificial intelligence
CVI: content validity index
WRP: weighted ranking points
